# The Impact of Climate Change on the Food System in Toronto

**DOI:** 10.3390/ijerph15112344

**Published:** 2018-10-24

**Authors:** Kimberly Zeuli, Austin Nijhuis, Ronald Macfarlane, Taryn Ridsdale

**Affiliations:** 1 Initiative for a Competitive Inner City, 56 Warren Street, Boston, MA 02119, USA; anijhuis@icic.org; 2 Formerly with Toronto Public Health, 277 Victoria Street, Toronto, ON M5B 1W2, Canada; RonaldM@mail2world.com; 3 Toronto Public Health, 277 Victoria Street, Toronto, ON M5B 1W2, Canada; Taryn.Ridsdale@toronto.ca

**Keywords:** climate change, extreme weather events, food systems, vulnerable populations

## Abstract

As part of its Climate Change and Health Strategy, in 2017, Toronto Public Health engaged stakeholders from across the food system to complete a high-level vulnerability assessment of the impact of climate change on the food system in Toronto. Using the Ontario Climate Change and Health Vulnerability and Adaptation Assessment Guidelines, the City of Toronto’s High-Level Risk Assessment Tool, and a strategic framework developed by the Initiative for a Competitive Inner City, Toronto Public Health identified the most significant extreme weather event risks to food processing, distribution and access in Toronto. Risks associated with three extreme weather events that are the most likely to occur in Toronto due to climate change were analyzed: significant rain and flooding, an extended heat wave, and a major winter ice storm. The analysis finds that while extreme weather events could potentially disrupt Toronto’s food supply, the current risk of an extended, widespread food supply disruption is relatively low. However, the findings highlight that a concerted effort across the food system, including electrical and fuel providers, is needed to address other key vulnerabilities that could impact food access, especially for vulnerable populations. Interruptions to electricity will have food access and food safety impacts, while interruptions to the transportation network and fuel will have food distribution and access impacts. Actions to mitigate these risks could include addressing food access vulnerabilities through ongoing city-wide strategies and integrating food access into the City’s emergency response planning. The next steps will include engaging with multiple partners across the city to understand and strengthen the “last mile” of food distribution and develop community food resilience action plans for vulnerable neighbourhoods.

## 1. Introduction

Climate change is expected to increase the severity and frequency of disruptive weather events [1,2] which pose a significant risk to urban food systems and ultimately the health of urban residents. Food and health are interconnected across the food system, from food production through food consumption and waste disposal.

Toronto is at the forefront of cities working to manage the risk of food system disruption. In its Climate Change and Health Strategy for Toronto, “A Climate of Concern”, Toronto Public Health (TPH) shared its concerns and recommended that the impact of climate change on food safety, security and sustainability should be analyzed [3]. To address this issue, TPH, in partnership with the Environment and Energy Division, completed a high-level climate change vulnerability assessment of Toronto’s food system with the assistance of the Initiative for a Competitive Inner City (ICIC), a non-profit research organization working at the forefront of urban food resilience. The assessment considered three extreme weather events: significant rain and flooding, an extended heat wave, and a major winter ice storm.

As cities formulate their response to climate change and extreme weather events, they often overlook urban food systems—i.e., the distribution and access of food within cities. Yet, hurricane flooding in New Orleans in 2005, and more recently in the Caribbean and Florida in 2017, caused significant food supply disruptions and suggests that this is a critical oversight [4,5,6]. Although Toronto is not likely to experience food supply disruptions of this same magnitude, extreme storms in the past have created localized food access issues for residents that have lasted up to a week. Climate change is expected to cause an increase in the severity and frequency of certain extreme weather events, which could cause extended food supply and access disruptions beyond the initial emergency response and recovery period.

**Figure 1:**
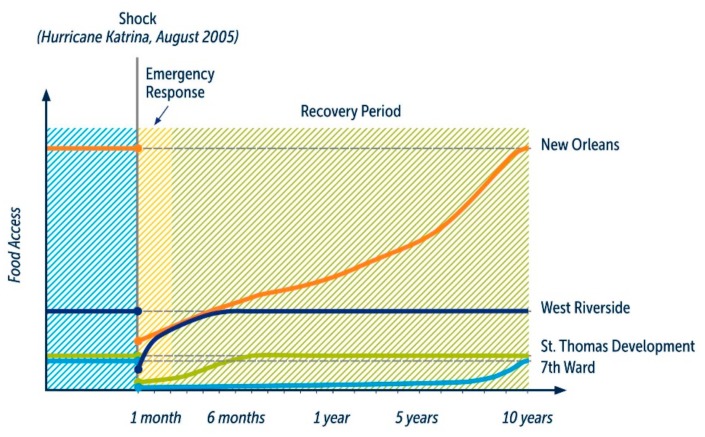
Food system recovery after hurricane Katrina.

The drive to create a more efficient food system, with “just-in-time” distribution, and food industry consolidation have created urban food systems that are fundamentally vulnerable to food supply disruptions. For example, researchers estimate that most food retail stores in Toronto may only have 3 days of fresh food and up to 17 days of all food products in stock [7,8]. Further, evidence from natural disasters in other cities, including New Orleans, shows that food system disruption at the neighbourhood level will vary because of differences in their food insecurity rates and food retail markets (Figure 1).

In this paper, we present the results from the vulnerability assessment of the city of Toronto’s food system to three extreme weather events. To complete the vulnerability assessment, we analyzed public and proprietary data, conducted interviews with 49 individuals, and facilitated a workshop with 23 stakeholders representing various aspects of Toronto’s food system. The analysis reveals that while certain aspects of Toronto’s food system would be quite resilient to extreme weather events, a concerted effort across the food system is needed to address five critical vulnerabilities.

## 2. Materials and Methods

Three complementary frameworks were used to shape the methodology: ICIC’s Framework for Analyzing Urban Food System Resilience, the Ontario Climate Change and Health Vulnerability and Adaptation Assessment Guidelines, and the City of Toronto’s High-Level Risk Assessment (HLRA) Tool. Three extreme weather events were analyzed: (1) flooding from an extreme rainfall event of 100 mm in less than 1 day; (2) a heat wave with 3 or more consecutive days when the maximum temperature is 35 degrees Celsius or higher; and (3) an ice storm causing 30 mm of ice. Toronto has already experienced each of these extreme weather events, and they are expected to become more frequent and severe in the future [9]. The areas in and around Toronto most likely to be affected by the event are considered “at risk” areas.

### 2.1. ICIC’s Framework for Analyzing Urban Food System Resilience

ICIC’s urban food system resilience framework is a streamlined approach to analyzing the resilience of food systems to different types of disasters. It is centered on the food system components within a metropolitan area that determine food availability (i.e., the supply of food that is available for purchase and consumption) and food access (i.e., the ability to purchase or access sufficient, preferred food). The components analyzed include food processing, food distribution, food retail and food access mechanisms (i.e., food banks). Other determinants of food security—food utilization and stability—are not included in the framework [10]. The framework’s neighbourhood level analysis of food availability and food access intentionally considers equitable resilience, where all residents have adequate access to food within walking distance immediately after an extreme weather event.

Almost all food consumed in cities is produced outside the conurbation, but some of it is processed locally. Because only a small share of food consumed in cities is produced locally, food production is excluded from the framework. Food production will, of course, also be impacted by extreme weather events and broader climate change (e.g., drought conditions and higher average temperatures), which will affect crop yields and the prevalence of pests and diseases, and shift production patterns with changes in land and water suitability for crops [11]. In turn, this is likely to cause disruptions in the supply of fresh food and at food processing plants within metropolitan areas. Given the global nature of food production that supports a city like Toronto, however, analyzing food production vulnerabilities was beyond the scope of our analysis.

### 2.2. Ontario Climate Change and Health Vulnerability and Adaptation Assessment Guidelines

The Ontario Ministry of Health and Long-Term Care developed the Ontario Climate Change and Health Vulnerability and Adaptation (V&A) Assessment Guidelines in 2016 to provide public health units across Ontario with a practical toolkit for conducting comprehensive vulnerability and adaptation assessments to climate change risks [12]. The primary objectives of the V&A assessment guidelines are to understand the current and projected future public health risks of climate change and to identify and develop policies and programs to increase resilience to these risks. The structure of the V&A guidelines are designed to be flexible and can be tailored to the circumstances of the assessment.

### 2.3. The City of Toronto’s High-Level Risk Assessment Tool

The City of Toronto’s High-Level Risk Assessment (HLRA) tool is used to solicit input from a diverse group of key stakeholders through a structured workshop. This tool represents a distillation of Toronto’s more complex and in depth Climate Risk Assessment Tool, which was originally developed to mirror the International Organization for Standardization (ISO) 37000 Risk Management standard. The City of Toronto developed the HLRA tool to help implement its Climate Change Risk Management Policy, which was designed to evaluate the ability of the city’s infrastructure to accommodate extreme weather events. As of 2017, the HLRA tool had been used to assess the resilience of three different “Thematic Areas”: utilities, transportation and water.

### 2.4. Points of Vulnerability Analyzed in Toronto’s Food System

Taken together, the three frameworks identified points within the food system to be analyzed (Table 1). Data and data resources for the Toronto Vulnerability Assessment were showed in Table S1. The food system is an interconnected system, meaning its functioning depends upon the performance of numerous other systems in a city. The most critical interdependencies include public transportation; the road network; the electrical power system; telecommunications; and the fuel supply transportation, storage and distribution infrastructure. These were incorporated into the analysis. In addition, the analysis also explored the prevalence of risk management tools (business continuity plans and insurance) being used by private-sector food companies and non-profit organizations to manage some of the risks of extreme weather events. Finally, the analysis included Toronto’s municipal policies and procedures that could affect food system recovery.

### 2.5. Extreme Weather Event Assumptions

The risk of flooding in Toronto is caused by several elements, including but not limited to, precipitation, proximity to water bodies, topography, soil type, and land use [13]. Toronto lies along Lake Ontario. In addition, three larger rivers, the Humber River, the Don River, and the Rouge River, as well as numerous other smaller rivers and streams, run through the city and into Lake Ontario. A few areas in the city are prone to river-based (or riverine) flooding. A greater potential risk facing the city is “urban flooding,” also known as pluvial or overland flooding, which happens when extreme rainfall overwhelms sewer systems or when there is major overland water flow in low-lying areas [13]. “Urban flooding” is more complex to model than river-based flooding, and has not yet been fully modelled for Toronto.

**Figure 2:**
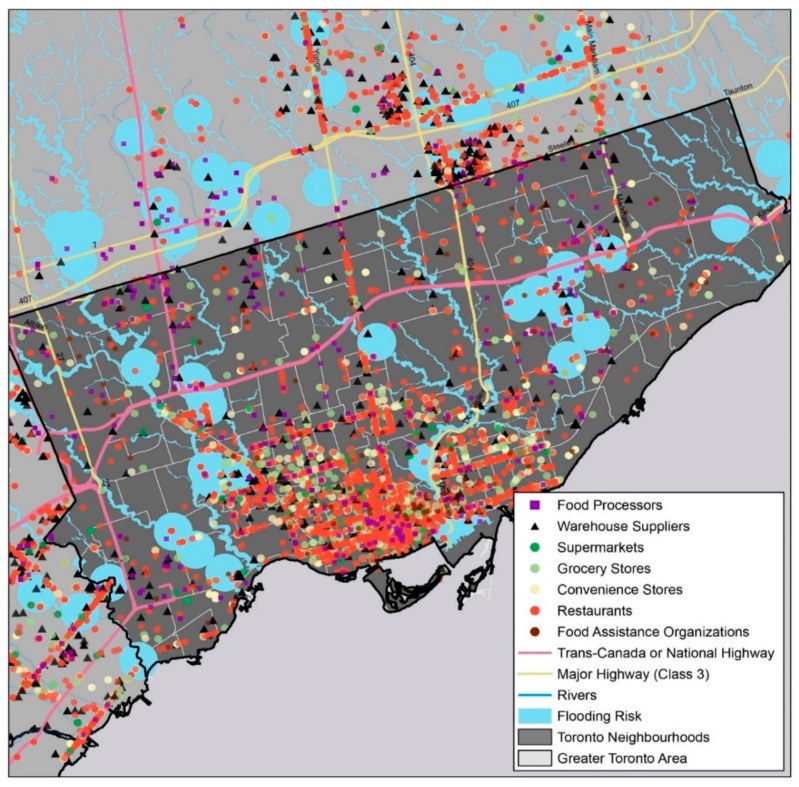
Riverine flood risk for Toronto’s food system. Sources: Canadian Food Inspection Agency, Registered Shell Egg Stations (2018); City of Toronto Social Development, Finance & Administration Division, Neighborhoods (2014); Dun and Bradstreet’s Hoovers Database (2017); Ontario Food Terminal Board, Directory: Ontario Food Terminal Board (n.d.); Ontario Ministry of Agriculture, Food and Rural Affairs, Provincially Licensed Meat and Dairy Plants (2017); Toronto and Region Conservation Authority, Regulator Flood Plain, Estimated Flood Plain, and Flood Vulnerable Areas (n.d.). Notes: Food processors include dairy processing facilities, poultry processing facilities, egg processing facilities, and commercial bakeries.

For this analysis, only readily available riverine-based flood maps were used. The areas considered vulnerable to river-based flooding from an extreme rain event are those that are either (1) in the Toronto and Region Conservation Authority (TRCA)’s regulatory (engineered) flood plain; (2) in TRCA’s estimated flood plain; or (3) in TRCA’s Flood Vulnerable Areas (Figure 2).

Heat waves in Toronto are defined by Environment and Climate Change Canada as 3 or more consecutive days when the maximum temperature is 32 degrees Celsius or more [14]. For this analysis, we assumed a heat wave with 3 or more consecutive days when the maximum temperature is 35 degrees Celsius or higher, as maximum temperatures in Toronto can exceed 35 degrees Celsius currently and the number of days when the maximum temperature is 35 degrees Celsius or higher is projected to increase because of climate change [9]. Ice storms are freezing rain events that form a coating of ice on the ground and on exposed objects [15]. We assumed an ice storm causing 30 mm of ice because Toronto has already experienced a similar event in December 2013 [16]. Since both heat waves and ice storms tend to be regional events, for the analysis it was assumed that anything located in Toronto or the Greater Toronto Area (GTA) is at risk for these extreme weather events.

## 3. Results

The following section summarizes the potential impact the three selected extreme weather events could have on Toronto’s food system. It includes both the direct impact, which is likely to be minimal in most cases, as well as the potentially more significant disruption caused by failures in critical infrastructure.

### 3.1. Food Processing Vulnerabilities

Because of the global nature of the food system, a very small share of total food consumed in any city is processed and packaged locally [17,18]. Therefore, food processing overall is not typically a critical area of vulnerability to a localized extreme weather event. The exceptions are dairy and bakeries, which are generally located near their customers due to the perishability of their products. In Toronto, a higher share of dairy, eggs and poultry products (chicken and turkey) that are consumed within the city are processed locally because of Canadian supply management regulations [19].

Six percent of dairy processing facilities in Toronto and the surrounding region and 10 percent of commercial bakeries are located in areas at risk for riverine flooding. No poultry processing facilities or egg processing facilities are located in areas at risk for riverine flooding.

Critical infrastructure failures would affect all of the facilities. Road closures or traffic congestion would disrupt their ability to source inputs and deliver products and would also affect employees who drive to work (making their commute longer or perhaps preventing their ability to reach the facility). Public transportation disruptions, however, might be a greater concern because it could potentially impact a larger number of their employees who might not have other means of getting to work. Power outages and telecommunication disruptions could also disrupt food supply, as raw ingredients and food would perish (loss of inventory), since processing equipment and coolers would not function without backup power. Orders also would need to be filled manually, taking significantly more time to complete. No data exists on how many dairy processing facilities and commercial bakeries have backup generators, but experts indicate that it is likely that most poultry and egg processing facilities have backup generators.

The risk with backup generators is that they would ultimately require more fuel (natural gas or diesel) to operate over an extended period. For facilities with backup generators powered by natural gas, some may have direct pipeline connections. For these types of generators, running out of fuel is less of a concern. For facilities with backup generators powered by diesel, diesel is delivered by truck and stored onsite. Facilities with these types of generators would need to be able to receive fuel deliveries by truck to continue to operate their backup generators. In addition, for facilities located in flood-prone areas, backup generators in low-lying areas (e.g., basements or at ground-level) increase the risk of failure as these generators may flood, potentially making them inoperable.

### 3.2. Food Distribution Vulnerabilities

One of the five primary distributors for supermarkets is located in an area vulnerable to riverine flooding. Of the 1489 local distributors that serve all food retail outlets and restaurants, 168 (11 percent) are located in areas at risk for riverine flooding.

Critical infrastructure failures would affect all distribution facilities in the same way they would affect food processing facilities. In addition, fuel shortages or limited access to fuel could also disrupt their ability to deliver product.

Power outages and telecommunication disruptions could also create the same type of supply disruption as discussed for food processing facilities. However, according to experts, most distribution centres for Toronto supermarkets have backup generators that would be able to fully power facilities and all equipment, although they would face the fuel supply risks discussed above.

Large food retailers typically require their distributors to have business continuity plans in place as part of their purchasing contracts. Large food retailers also may work directly with suppliers to improve their business continuity planning, which helps reduce their vulnerabilities.

Food distribution networks have some inherent resilience because they are typically fragmented, with distributors spread across various locations. National and vertically integrated distributors are also well positioned to handle disruptions because they operate in multiple locations and have the resources to invest in structural improvements to withstand disasters, including sufficient backup power. Smaller, local distributors are less likely to have business continuity plans in place, generally operating in only one location, and are less likely to invest in making their facilities more resilient to disaster [4,20]. Interviews with Toronto food distribution industry experts suggest that this holds true for Toronto’s food distribution networks. This creates a greater likelihood of supply disruptions to the food retailers they serve—mostly smaller grocery and convenience stores.

#### Ontario Food Terminal

Toronto has a large, centralized fresh produce distribution centre, the Ontario Food Terminal, which is the largest wholesale fruit and produce distribution centre in Canada, and the third largest in North America. It comprises 21 wholesalers and 400 farmer market tenants, who supply produce to over 5000 retailers across the country [21]. According to Ontario Food Terminal estimates, the Terminal distributes between 35 to 40 percent of produce sold in Toronto.

The Ontario Food Terminal is located in an area at risk for riverine flooding. According to a representative from the organization, they are less concerned about the impact of flooding on their property, which they believe would be minimal, but are more concerned about the potential impact on the Terminal’s electricity. The Terminal has only one transformer station that supplies power to its facilities and there is no redundancy for this transformer. The Ontario Food Terminal has a business continuity plan in place in the event of a power outage but it does not have a permanent backup generator. The Terminal does, however, carry flood and business interruption insurance and all tenants also carry business interruption insurance, which manages some of their business risks and will help them get back online quickly, but it does not ensure that fresh food supply would not be disrupted in the short-term.

Ice storms and heat waves could also lead to power outages at the Terminal. The December 2013 ice storm led to a power outage lasting 72 h. The Ontario Food Terminal was not able to sell produce to its buyers during this period [22]. The Terminal was able to preserve cold storage for products in storage due to the winter cold temperatures, but a more prolonged power outage, especially if it happened in warmer weather, would have meant their cold storage would likely have been compromised.

Road closures or traffic congestion due to extreme weather would disrupt the ability of warehouse suppliers located in the Ontario Food Terminal to receive or deliver products and would also make it difficult for buyers to access the facility. Since all food products in the Terminal are transported by trucks, fuel shortages or limited access to fuel would also disrupt the movement of fresh food in and out of the Terminal. Road and public transportation disruptions would also affect the ability of employees to get to work.

### 3.3. Food Retail Vulnerabilities

The analysis finds 22 out of 140 neighbourhoods (16 percent) in Toronto that have vulnerable food retail markets. Seventeen of the 22 neighbourhoods have no food retail stores. Vulnerable food retail neighbourhoods are those that either have no food retail stores, or those that meet the following criteria: (1) fewer food retail stores per capita than the city average (i.e., the neighbourhood is underserved); (2) the share of supermarkets is lower than the city average (i.e., neighbourhood residents rely more on smaller grocery and convenience stores for daily food needs); and (3) more than 50 percent of all food retail stores are located in areas at risk for riverine flooding.

According to Canadian food retail industry experts and representatives from food retailers interviewed for our analysis, the majority of supermarkets are likely to have short- and long-term business continuity plans in place. Smaller grocery stores may have short-term business continuity plans, but may not be prepared for long-term supply chain disruptions. Smaller grocery stores and convenience stores that are independently owned, and are not part of a national or regional chain, may not have adequate (or any) business continuity plans in place and may face longer periods of closure after an extreme weather event because they have limited access to supply chains, fewer resources and often insufficient (or no) insurance [20]. Even if they have insurance, owners of smaller food stores typically need to cover all costs associated with reopening their business while waiting for reimbursement from their insurance companies and assistance from public agencies. For some business owners, these costs can be prohibitive and they simply may not have the resources to reopen. In addition, the approval process for public disaster recovery funds is often slow and the distribution of funds for business owners can be delayed.

Critical infrastructure failures could impact all food retail stores. Road closures or traffic congestion would disrupt food deliveries from warehouse suppliers, customer access, and the commute time of employees. Public transportation disruptions might also impact customer access and the commute time of employees, especially for those who do not own cars. Power outages would impact cold storage, potentially causing some food to perish. Power outages combined with telecommunications disruptions would impact payment and ordering systems. As a result of these issues, most food retail stores close during power outages.

There is disagreement among the experts interviewed for this study about the prevalence of backup generators in Toronto’s supermarkets, grocery stores and convenience stores. There is no regulatory requirement for backup power in retail stores and no data on backup power trends among retailers.

### 3.4. Food Access Vulnerabilities

Food access is just one component of food security, which is also a function of economic conditions (i.e., affordability and income), food utilization (i.e., adequate diet) and stability [10]. All determinants of food security could be impacted by an extreme weather event and household food insecurity may be exacerbated for households who are already food insecure. We consider food insecure populations as those that are most vulnerable to food-related impacts from an extreme weather event. In the Toronto region, one in eight households are food *insecure* (defined as a household with inadequate or insecure access to adequate food due to financial constraints), which is roughly the same as the national average [23]. Neighbourhoods with both food retail vulnerabilities and high rates of food insecurity are at greatest risk of limited food access in the aftermath of an extreme weather event. To analyze food insecurity in the 22 neighbourhoods identified as having vulnerable food retail markets, household income and social assistance rates were used as proxies because food insecurity data at that geography is not available. While these are not perfect proxies for food insecurity (e.g., many households that are food insecure may not receive social assistance), these are the most reliable data available that can be used to gain some insight into neighbourhood level food insecurity.

The first step in the neighbourhood vulnerability analysis was to identify neighbourhoods where the share of population classified as low income is greater than the city average. This analysis reveals that 11 of the 22 vulnerable food retail neighbourhoods include a greater share of residents living on low-income than the city average. Within this set of 11 neighbourhoods, seven neighbourhoods have higher social assistance rates than the city average. This set of seven neighbourhoods are at greatest risk for limited food access after an extreme weather event because they have higher rates of food retail vulnerability and food insecurity. All of the seven neighbourhoods are in the inner suburbs, an area of Toronto that has experienced increased population growth, declining average income levels, and increased demand on food banks over the past decade.

#### High-Rises Pose a Challenge to Food Access Post-Disaster

Many factors can increase a population’s vulnerability to food insecurity. Populations living in high-rise buildings—especially those with limited mobility (e.g., older adults)—may be more vulnerable to food insecurity after an extreme weather event. Other factors beyond high-rise buildings and limited mobility (such as social cohesion and martial status) may also contribute to increased food insecurity vulnerability after an extreme weather event [24]; however, these factors are beyond the scope of this study.

Toronto is home to 493,270 high-rise apartment units, which accounts for 44 percent of all occupied private dwellings in the city. Many high-rises in the city are over 30 stories high, and the tallest residential high-rise is 78 stories [25]. Without working elevators, residents living on higher floors would have a more difficult time accessing food in the event of a power outage. Using the stairs would effectively increase the distance they would have to travel and would make it more difficult to carry food to their apartments.

In 2017, the City adopted guidelines for backup power in multi-unit residential buildings, but most high-rise dwellings are only required to have backup generators to power elevators and life safety systems (e.g., fire alarm system, standpipe and hose system, or sprinkler system) for up to 2 h during a power outage [26]. The backup generators are only meant to enable residents to evacuate their apartments and emergency services personnel to access the building. Older high-rise buildings can have particular vulnerabilities because many residents living in these buildings are on low incomes, newcomers to the city, or older adults (65 years and over). In Toronto, 39 percent of older adults live in high-rise apartments.

A comprehensive study on potential food deserts in high-rise neighbourhoods created by a lack of food retail access as well as reliance on electricity to power elevators, was recommended in a case study of resilience planning in Toronto [27]. A study on walkability in eight of Toronto’s high-rise neighbourhoods found that walking was the most common way for residents to access food [28].

Of the 22 neighbourhoods identified as having vulnerable food retail, six have a higher share of high-rise apartment dwellings compared to the city average. All six of these neighbourhoods also have a higher share of older adults compared to the city average. Of the seven neighbourhoods with both vulnerable food retail and higher rates of food insecurity, three have a higher share of high-rise apartment dwellings compared to the city average and a higher share of older adults compared to the city average.

### 3.5. Food Banks

A network of charitable food assistance organizations, which include food banks, soup kitchens and other hunger assistance programs, help food insecure households access more food. Although food assistance organizations were created to help people in need during times of severe financial constraint, they are supporting those in need for longer periods than intended.

Nine percent of Toronto’s 96 food banks are operating in facilities located in flood risk areas. According to a representative from Food Banks Canada, most food banks have flood insurance because they rent their facilities and their landlords would carry the policy, but very few would have business continuity plans.

Critical infrastructure failures could impact all of Toronto’s food banks. Road closures or traffic congestion would disrupt their ability to receive and deliver donated products, thereby creating some food supply disruptions to smaller food banks and other food assistance agencies. Fuel shortages or limited access to fuel would also disrupt the ability to deliver donated products. Road or public transportation disruptions would affect the ability of employees, volunteers and member agencies to get to the food banks. Road and public transportation disruptions would also affect the ability of individuals to access food banks.

Power outages and telecommunication disruptions would also create supply disruptions, as food in cold storage could perish. This would be a bigger issue during winter ice storms, when food banks receive a large supply of donated products during the holiday period.

Perhaps the greater risk of an extreme weather event is that Toronto’s food banks might not be able to meet a prolonged increase in demand for food assistance. Previous studies have shown that in the aftermath of an extreme weather event, demand for food from food assistance organizations remains elevated for a prolonged period [4]. If these organizations do not have the capacity or resources to meet current demand, they will be less likely to meet increased demand, especially over an extended period.

A recent study found that most food assistance agencies in Toronto are not fully meeting their clients’ food needs. Specifically, 78 percent of agencies reported that their clients needed more food than the agency was able to provide, 62 percent of agencies sometimes altered the variety of food provided due to a lack of food, and 53 percent of agencies sometimes cut the size of food hampers provided because of insufficient food [29].

### 3.6. Restaurant Vulnerabilities

Many Toronto residents rely on restaurants for a substantial share of their daily food needs. In Ontario, 33 percent of the average household’s food expenditures are spent on food purchased from restaurants, according to the 2015 Survey of Household Spending data from Statistics Canada. There are 6096 restaurants in Toronto and 275 (5 percent) are located in areas at risk for riverine flooding. In five of the 22 neighbourhoods with vulnerable food retail, 50 percent or more of the neighbourhood restaurants are located in areas at risk for riverine flooding, making them even more vulnerable to food access issues after an extreme weather event.

Critical infrastructure failures could impact all food restaurants. Fuel (natural gas) is needed for meal preparation. Road closures would disrupt deliveries from warehouse suppliers, impact employee commute time and could prevent some customers from patronizing the restaurants. Public transportation disruptions could also affect employee commute time and could prevent some customers from patronizing the restaurants, especially for those who do not own cars. Power outages would affect refrigerators and freezers, potentially causing food to perish. Power outages combined with telecommunications disruptions would impact payment and ordering systems. Experts interviewed for this study believe that most restaurants do not have backup generators.

Restaurants may not have the financial resources or insurance to prepare for an extreme weather event [20]. According to an Ontario restaurant industry expert interviewed for our analysis, restaurants do not prioritize purchasing backup generators, or flood or business interruption insurance. In addition, for restaurants that rent their space, it is often the building owner who would be responsible for purchasing and installing backup generators and purchasing flood insurance. In Toronto, 82 percent of restaurants rent their space.

### 3.7. Home Meal Preparation and Storage

Prolonged power outages can lead to bacteria build-up on perishable food stored in refrigerators and freezers, making them unsafe to eat. Toronto Public Health food safety guidelines state that food will keep for 24 to 48 h in a freezer and for 12 to 24 h in the fridge during and after an extended power outage, provided the doors remain closed [30]. A more prolonged power outage means that most perishable food would become unsafe to eat.

A loss of power also limits the ability of households to prepare hot meals and may lead to improper food handling. Further, domestic water supply systems above the sixth or seventh floors in high-rises would probably not be operational during power outages because booster pumps are required for getting water above this level.

After a disaster with extended power outages, Toronto Public Health releases public reminders about food safety guidelines. In addition, inspectors conduct regular visits to all City emergency reception centres during and after a disaster to ensure food safety protocols are in place [31].

### 3.8. Government Policies and Practices

In Canada, federal, provincial, and municipal governments are responsible for inspecting all food facilities (any facility that touches food) to verify that food sanitation practices are being followed [32]. If an extreme weather event causes any food facility, including warehouse suppliers, food retail stores or restaurants to voluntarily close, they do not need to pass a food safety inspection to reopen [33,34]. Toronto Public Health inspectors will conduct spot checks of food facilities to ensure food is safe for sale. Toronto Public Health inspectors will prioritize inspections in areas hardest hit by an extreme weather event and facilities most vulnerable to food safety issues, such as hospitals, daycare facilities and full-service restaurants. In a widespread event, Toronto Public Health would seek assistance from inspectors in other departments, from surrounding health units, and from Ontario Ministry of Agriculture, Food and Rural Affairs.

Various municipal and provincial government agencies will need to be actively engaged in helping the food system recover quickly after an extreme weather event, which makes efficient coordination and communication challenging. Multiple non-governmental organizations (NGOs) would also provide emergency response and recovery support after an extreme weather event in Toronto. Toronto has an emergency food plan in place to cover the distribution of food and water in the immediate aftermath of any disaster, which includes a coordinated multi-agency response [35].

However, the food system stakeholders that informed this report expressed concerns about the government’s preparedness planning, citing the need for private sector participation in the government’s planning process, and worries that there would not be clear communication between government and private sector organizations in the aftermath of an extreme weather event.

## 4. Discussion

This analysis makes an important contribution to the City’s climate resilience planning by providing an overall assessment of the vulnerability of Toronto’s food system to extreme weather events and by highlighting strategic vulnerabilities that should be addressed to ensure equitable food access across Toronto after such a disaster. Five significant vulnerabilities were identified:

*Flooding*: The impact of an extreme rain event is the least well understood compared to extreme heat and an ice storm. Only riverine flooding was included in our analysis because “urban flooding” has not yet been fully modelled for the city. Given the research to date, however, river and urban flooding pose the greatest risk of the three extreme weather events studied for the food system. Urban flooding is a recognized risk in Toronto that affects areas outside of the riverine flood plains. Better understanding the areas at risk of urban flooding would contribute to better risk mitigation and emergency response plans.

*Electricity, Road Network and Fuel Infrastructure*: The food sector is highly dependent on different forms of infrastructure and electricity; roads, and fuel supply are the most significant points of vulnerability.

*Food Access in Vulnerable Neighbourhoods*: Food access in 22 of Toronto’s neighbourhoods is disproportionately at risk from extreme weather events because of limited and vulnerable food retail stores. Seven of the 22 neighbourhoods are at greatest risk for limited food access after an extreme weather event because they also have higher rates of food insecurity. In some of these neighbourhoods, vulnerable populations living in older high-rises could be significantly impacted, particularly people with low mobility.

In the aftermath of an extreme weather event, access to food can have an important role in maintaining or enhancing social cohesion [24]. While more studies are needed to better understand the factors involved, social cohesion can foster quicker recovery in the aftermath of an extreme weather event. These findings suggest that cities need to prioritize planning for neighborhoods most vulnerable to extreme weather events, taking into account vulnerable populations, where food access could be severely constrained for an extended period of time. Developing local community food resilience action plans and food emergency response plans in neighbourhoods with critical food access issues can help address food insecurity and increase the community’s emergency food preparedness, response and recovery.

*The Food Assistance Network*: Food insecurity is a systemic vulnerability in Toronto that can be exacerbated by extreme weather events and is a critical consideration for equitable resilience strategies. Although food banks and other food assistance organizations were created to help people in need during times of severe financial constraint, they are supporting those in need for longer periods than intended. Many food banks rely on increased monetary or food donations to meet temporary increases in demand for food assistance immediately after a disaster, and are, therefore, able to provide temporary assistance. Their ability to meet a prolonged increase in demand for food assistance as more households become food insecure due to disaster-related expenses or loss of income is less certain as they experience donor fatigue.

*Coordination, Planning and Preparedness*: Coordination and communication across government agencies and with NGOs and private sector food companies will be challenging in the aftermath of an extreme weather event. More may need to be done by the City, non-profit organizations and private sector businesses to prepare for these situations and to develop more effective protocols. Preparedness planning needs to include the right representatives from across the food system.

## 5. Conclusions

This study identified several climate-related vulnerabilities of the food system in Toronto, but also more broadly adds to the growing body of research on urban resilience. It shows the importance of analyzing and addressing vulnerabilities in the food system related not only directly to climate change, but also to extreme weather events that are likely to become more intense and frequent with climate change. Very few studies have explored food system resilience and fewer still have integrated a public health perspective that includes food insecurity and food safety issues. A body of research already exists that analyzes food safety issues that arise from extreme weather events, such as water contamination and foodborne disease outbreaks from improper food storage and unsafe food preparation during power outages; however, this work has not yet been integrated into food system resilience studies. A deeper integration is warranted, but beyond the scope of this study. The methodology and insights from this study are relevant for cities globally, but more comparative research needs to be done across different urban food systems.

This study provides additional evidence that shows the reliance of urban food systems on transportation, energy, and telecommunication infrastructure, and highlights the need to address vulnerabilities in supporting infrastructure as a leading disaster risk mitigation strategy for reducing food system disruption as a result of extreme weather events. In Toronto, interruptions to electricity could create the most significant food access and food safety impacts, while interruptions to the transportation network and fuel could significantly disrupt food distribution. In addition to their current household-level interventions, public health practitioners and policy makers should begin to address these underlying food system risks at the city level. This study also demonstrates that city-wide resilience building strategies that mitigate the risk of extreme weather events are not sufficient. Specific areas (and populations) within a city will be disproportionately impacted by food system disruptions. People who are already food insecure will be most affected. Thus, cities also need to prioritize neighbourhood-level food resilience plans for neighbourhoods most vulnerable to extreme weather events, taking into account their most vulnerable populations. In the long-term, risk mitigation will require policy initiatives that address food insecurity, including investing in building more supermarkets and other healthy food access mechanisms in vulnerable neighbourhoods to improve food access and availability, as well as developing robust emergency response plans. In the short-term, integrating food access into the Toronto’s neighbourhood emergency response plans, making sure that food banks have sufficient resources to meet increased demand and that supermarkets, grocery and convenience stores have adequate business continuity plans, insurance and back-up power, should be risk mitigation priorities. The food retail industry will need to be engaged to support these strategies.

Our methodology provides a streamlined approach for cities interested in identifying where vulnerabilities might exist in their food system. Thus, additional studies into specific vulnerabilities are a necessary next step in risk mitigation to identify specific risks that will point to specific mitigation strategies. Further, more comprehensive studies are needed in cities to fully analyze the production points and the flow of all food products, including the “last mile,” to increase food resilience at both neighbourhood and city-wide levels to both extreme weather events and global climate change.

The complete assessment, Resilient Food Systems, Resilient Cities: A High-Level Vulnerability Assessment of Toronto’s Food System, is posted on ICIC’s website [36].

## Figures and Tables

**Table 1:** Approach for the Toronto Vulnerability Assessment.

Points of Vulnerability	Approach for Analysis
Food processing	Analyze location of processing plants for: (1) dairy; (2) eggs; (3) chicken; and (4) turkey; as well as (5) commercial bakeries in “at risk” areas.
Food distribution	Analyze location of (1) primary warehouse suppliers (also known as wholesalers or distribution centres) for the city’s supermarkets; and (2) secondary suppliers within the city that move food from processing facilities to food retail stores and other food access points (e.g., restaurants, food banks, etc.) in “at risk” areas. This includes the Ontario Food Terminal. For all companies; analyze (3) business continuity plans; and (4) adequate insurance.
Food retail	Food retail stores include supermarkets, grocery stores and convenience stores. (1) Compare food retail stores per capita in each neighbourhood with city average, (2) compare share of supermarkets in each neighbourhood with city average; (3) measure share of food retail stores in each neighbourhood that are located in “at risk” areas; (4) analyze food retail business continuity plans and (5) adequate insurance coverage by food retail stores.
Food insecurity	Compare share of low-income residents and social assistance recipients in each neighbourhood to city average.
Food banks	Analyze (1) location of food bank in “at risk” areas; (2) ability of food bank to meet current demand; (3) food bank plans to meet increased demand over extended period of time; (4) food bank business continuity plans, and (5) adequate insurance coverage by food banks.
Restaurants	Analyze location of restaurants in “at risk” areas.
Home meal preparation and storage	Analyze standard meal preparation and storage guidelines and ability to meet them after an extreme weather event, including in high-rise apartment units.
Public transportation	Trains, subways, buses and streetcars that allow Toronto residents to access food or workers in the food sector to commute to work. Analyze location of subway stations in “at risk” areas and (1) exposure of train, subway, streetcar and bus routes to extended closure and (2) continuity plans of the public transportation system based on interviews with public transportation experts and review of previously completed public transportation risk assessments. Continuity plans include backup power, storm drainage pumps and ability to reroute or replace service.
Road network	Trans-Canada or National highways, major highways, secondary highways (major streets and arterial roads), collector roads, local roads, bridges and culverts used to distribute food to retail stores in Toronto and allow residents to access food. Analyzed (1) redundancy of highways used for transporting food into and within city and (2) exposure of food transportation routes to extended closures.
Electrical power system	The system of transmission terminal stations, municipal substations, switches, transformers and overhead and underground wires used to provide electrical power to residential, commercial, and industrial customers. Analysis of (1) electrical power subsystems located in “at risk” areas and (2) exposure of electrical power system to extended power outages based on interviews with electrical power system experts and review of previously completed electrical power system risk assessments.
Telecommunications	The network of land, mobile phones and internet service over which communications are transmitted. Analysis of (1) exposure of telecommunications to extended outages and (2) dependency of telecommunications on the electrical power system and fuel supply transportation, storage and distribution based on interviews with telecommunications experts.
Fuel supply transportation, storage and distribution	All infrastructure required to process, transport, store, and distribute liquid fuels. Liquid fuels relevant to the food system include gasoline, diesel, propane and natural gas. Analysis of exposure of fuel supply transportation, storage and distribution to shortages and disruptions.
Government policies and practices	Analysis of post-disaster (1) food safety inspection process; (2) transportation restrictions for food distribution trucks; (3) communication; and (4) analysis of preparedness planning with the private sector.

## Supplementary Materials

The following are available online at http://www.mdpi.com/1660-4601/15/11/2344/s1, Table S1: Data and Data Sources for Toronto Vulnerability Assessment.

## References

[1] American Meteorological Society (2018). Explaining Extreme Events of 2016 from A Climate Perspective.

[2] National Academies of Sciences, Engineering, and Medicine (2016). Attribution of Extreme Weather Events in the Context of Climate Change.

[3] Toronto Public Health (2015). A Climate of Concern: Climate Change and Health Strategy for Toronto.

[4] Zeuli K., Nijhuis A. (2017). The Resilience of America’s Urban. Food Systems: Evidence from Five Cities.

[5] Arnold K. Hurricane Irma Challenges Florida Grocery Pipeline. https://www.orlandosentinel.com/business/consumer/os-hurricane-irma-grocery-supply-chain-20170919-story.html.

[6] Ahmed A., Semple K. A Devastated Island’s Cry: ‘All the Food Is Gone’. https://www.nytimes.com/2017/09/10/world/americas/irma-caribbean-st-martin.html.

[7] Medical Officer of Health (2008). Proposal for Development of a Toronto Food Strategy.

[8] Bristow D.N., Kennedy C.A. (2013). Urban metabolism and the energy stored in cities. J. Ind. Ecol..

[9] SENES Consultants Limited (2011). Toronto’s Future Weather and Climate Driver Study.

[10] FAO Agriculture and Development and Economics Division (2006). Food Security.

[11] Government of Canada (2014). Canada in a Changing Climate: Sector Perspectives on Impacts and Adaptation.

[12] Ebi K., Anderson V., Berry P., Paterson J., Yusa A. (2016). Ontario Climate Change and Health Vulnerability and Adaptation Assessment Guidelines: Technical Document.

[13] Flood Plain Map. https://trca.ca/conservation/flood-risk-management/flood-plain-map-viewer/.

[14] Weather Glossary. https://www.ec.gc.ca/meteoaloeil-skywatchers/default.asp?lang=EN&n=7884CDEA-1.

[15] Weather and Meteorology Glossary. http://www.ec.gc.ca/meteo-weather/default.asp?lang=En&n=B8CD636F-1&def=allShow#wsDT02BFC0DE.

[16] Toronto Ice Storm Leaves 230,000 without Power. https://www.cbc.ca/news/canada/toronto/toronto-ice-storm-leaves-230-000-without-power-1.2473543.

[17] Paxton A. (1994). The Food Miles Report: The Dangers of Long-Distance Food Transport. of Food.

[18] Weber C.L., Matthews H.S. (2008). Food-Miles and the Relative Climate Impacts of Food Choices in the United States. Environ. Sci. Technol..

[19] Heminthavong K. (2015). Canada’s Supply Management System. Brief, 2015-138-E.

[20] Runyan R.C. (2006). Small business in the face of crisis: Identifying barriers to recovery from a natural disaster. J. Contingencies Crisis Manag..

[21] Welcome to the Ontario Food Terminal Board. http://www.oftb.com/home.

[22] Ontario Ministry of Agriculture, Food and Rural Affairs (2017). Ontario Food Terminal—Three Year Business Plan. 2014–2017.

[23] Tarasuk V., Mitchell A., Dachner N. (2014). Household food insecurity in Canada, 2012.

[24] Clay L.A., Papas M.A., Gill K., Abramson D.M. (2017). Application of a Theoretical Model Toward Understanding Continued Food Insecurity Post Hurricane Katrina. Disaster Med. Public Health Prep..

[25] Pigg S. Toronto Leading the Western World in High-Rise Development. https://www.thestar.com/business/2012/12/12/toronto_leading_the_western_world_in_high_highrise_development.html.

[26] City of Toronto Environment & Energy Division (2016). Minimum Backup Power Guidelines for MURBs: Voluntary Performance Standards for Existing and New Buildings.

[27] Bristow D. (2015). Asset system of systems resilience planning: The Toronto case. Infrastruct. Asset Manag..

[28] Hess P., Farrow J. (2011). Walkability in Toronto’s High.-Rise Neighbourhoods.

[29] Tarasuk V., Dachner N., Hamelin A.-M., Ostry A., Williams P., Bosckei E., Poland B., Raine K. (2014). A survey of food bank operations in five Canadian cities. BMC Public Health.

[30] Toronto Public Health (2009). Power Failure and Food Safety.

[31] Scheuer K. (2013). Food Safety during Toronto’s Ice Storm and Power Outage.

[32] Canadian Food Inspection Agency (2017). Restaurant and Food Service Inspection in Canada.

[33] Province of Ontario (2017). Health Protection and Promotion Act..

[34] City of Toronto (2005). Toronto Municipal Code.

[35] City of Toronto Office of Emergency Management (2016). City of Toronto Emergency Plan. Emergency Support. Function: Incident Management System (IMS).

[36] Zeuli K., Nijhuis A., Gerson-Nieder Z. Resilient Food Systems, Resilient Cities: A High-Level Vulnerability Assessment of Toronto’s Food System. http://icic.org/wp-content/uploads/2018/07/ICIC_Toronto-Food-System_FINAL.pdf.

