# Supplementary material for: The Impact of Climate Change on the Food System in Toronto
Source: Int J Environ Res Public Health. 2018 Oct 24;15(11):2344. doi: 10.3390/ijerph15112344 (PMC6266038; doi:10.3390/ijerph15112344)
Supplement: Supplementary file 1 [file ijerph-15-02344-s001.pdf]

The Impact of Climate Change on the Food System in Toronto
Supplement: Data and Data Sources

Table S1. Data and Data Sources for Toronto Vulnerability Assessment

Food System Sectors	Data	Data Source
Food processing	Dairy processing facilities in the Greater Golden Horseshoe: 98 (6% located in areas at risk for flooding)	2017 Ontario Ministry of Agriculture, Food and Rural Affairs (OMAFRA) list of provincially licensed dairy plants
	Poultry processing facilities in the Greater Golden Horseshoe: 28 (0 located in areas at risk for flooding)	2017 Ontario Ministry of Agriculture, Food and Rural Affairs (OMAFRA) list of provincially licensed meat plants
	Egg processing facilities in the Greater Golden Horseshoe: 26 (0 located in areas at risk for flooding)	2018 Canadian Food Inspection Agency registered shell egg stations
	Commercial bakeries in the Greater Golden Horseshoe: 492 (10% located in areas at risk for flooding)	2017 Dun and Bradstreet's Hoover's Database for NAICS code 311812 (Commercial Bakeries)
Food distribution	Primary distributors in Greater Toronto Area: 5 (20% located in areas at risk for flooding)	2017 Dun and Bradstreet's Hoover's Database and the Ontario Food Terminal's directory of warehouse tenants. We identified all businesses classified as NAICS code 4244 (Grocery and Related Product Merchant Wholesales) in 2017 Dun and Bradstreet's Hoover's Database and all businesses listed as warehouse tenants in the Ontario Food Terminal's directory of warehouse tenants.
	Local distributors in Greater Toronto Area: 1,489 (11% located in areas at risk for flooding)	

Food System Sectors	Data	Data Source
Food retail	Neighbourhoods with vulnerable food retail markets (varies by neighbourhood)	Food retail data is from 2017 Dun and Bradstreet's Hoover's Database and includes all businesses classified as NAICS code 445110 (Supermarkets and Other Grocery Stores, except Convenience Stores), NAICS code 445120 (Convenience Stores) and NAICS code 447110 (Gasoline Stations with Convenience Stores). Neighbourhoods are defined by the City of Toronto Social Development, Finance & Administration Division and are available through the Toronto Open Data catalogue. Neighbourhoods are based on Statistics Canada census tracts. There are 140 total neighbourhoods in the city of Toronto.
Food insecurity	Population in Toronto classified as low income (varies by neighbourhood)	Low income population and population data from Wellbeing Toronto. Low income population is from 2012, the most recent year available from Wellbeing Toronto. Total population is from 2011, the closest year to 2012 with available data. Low income population is defined by Statistics Canada as the population earning less than 50 percent of the median adjusted after-tax income of households (the Low-Income Measure After Tax [LIM-AT] threshold).
	Population in Toronto receiving social assistance (varies by neighbourhood)	Social assistance rate is defined as the share of the total neighbourhood population receiving social assistance from Ontario Works (OW) or Ontario Disability Support Program (ODSP). Our analysis uses 2012 social assistance population and population data from Wellbeing Toronto, the most recent year available.

Food System Sectors	Data	Data Source
	High-rise apartment units in Toronto (Toronto and by neighbourhood). Toronto: 493,270 high-rise apartment units (44% of all occupied private dwellings)	2016 Census data from Statistics Canada. We use the definition for high-rise dwellings used by the City of Toronto (five-or more stories).
	Seniors 65 years and over living in high-rise apartments (Toronto and by neighbourhood). Toronto: 39% of older adults 65 years and over live in high-rise apartments	2011 National Household Survey data from Statistics Canada.
Food banks	Food banks in Toronto: 96 (9% located in areas at risk for flooding)	Food banks include Ontario Association of Food Banks (OAFB) food bank members provided by OAFB, food bank members of Daily Bread Food Bank and North York Harvest Food Bank, and food banks listed on 211 Toronto.
Restaurants	Restaurants in Toronto: 6,096 (5% located in areas at risk for flooding)	2017 Dun and Bradstreet's Hoover's Database. Restaurants include all businesses classified as NAICS code 7225 (Restaurants and Other Eating Places)
	Restaurants in Toronto that rent: 82%	
